# A Missing Link between Neuron Specific Enolase Release and Poor Prognosis in Aging Patients with B-cell Lymphoma

**DOI:** 10.4172/2155-9899.1000e121

**Published:** 2018-05-15

**Authors:** Rachel Polcyn, Jason God, Mollie Capone, Denise Matzelle, Naren L Banik, Azizul Haque

**Affiliations:** 1 Department of Microbiology and Immunology, Hollings Cancer Center, Medical University of South Carolina, Charleston, USA; 2 Department of Neurosurgery, Medical University of South Carolina, Charleston, USA; 3 Ralph H. Johnson Veterans Administration Medical Center, Charleston, SC, USA

## Editorial

B-cell lymphoma is a potentially life-threatening malignant disease. In 2018, the American Cancer Society estimates that approximately 83,180 people will be diagnosed with B-cell lymphoma, and 20,960 people will die from the disease [1]. Low grade B-cell lymphomas are slow-growing and often responsive to treatment; therefore, patients with low grade B-cell lymphoma can usually live for several years after their diagnosis. Conversely, high grade B-cell lymphomas are aggressive, fast growing, and require an intensive treatment plan. The specific treatment regimen varies depending on the type of lymphoma and severity of each individual case, but traditional methods, such as chemotherapy, surgery and radiation therapy have several drawbacks that necessitate further exploration of other, more tolerable methods. Recently, immunotherapy has been successfully used alone or in combination with chemotherapy, radiation and surgery to treat some lymphoma patients [2]. However, many late stage lymphoma tumors escape immune recognition by mechanisms that remain unknown. Our recent study suggests that B-cell lymphomas secrete inhibitory molecules that disrupt immune recognition of tumors [3]. We and others have also shown that aggressive B-cell tumors express or secrete factors that may be linked with poor prognosis for lymphoma patients. Because demographics in the US forecast a large, sustained increase in this group of malignancies in the near future, further understanding of the mechanisms by which these tumors escape immune surveillance is greatly warranted. This editorial outlines the possible link between B-cell lymphoma and neuronal enolase, a multifunctional enzyme known to exhibit pro-inflammatory or inhibitory activities, in an attempt to further excavate tumor evasion mechanisms in B-cell lymphoma.

B-cell lymphomas are mainly sub-classified into two types: (a) Hodgkin’s Lymphoma (HL) and (b) Non-Hodgkin’s Lymphoma (NHL). While HL causes serious malignancy in children and adults, NHL is the 6th most common cause of cancer death in both men and women in the United States, accounting for approximately 90% of all lymphoma cases and 4% of all cancer cases in the US [4]. NHL primarily affects B-cells (80–85% of all NHL) though it can also be derived from T cells or NK cells. Although the incidence and mortality of most cancers are decreasing, the incidence and mortality of NHL is increasing [5]. Common types of NHL include Diffuse Large B-Cell Lymphoma (DLBCL), Burkitt Lymphoma (BL), Follicular Lymphoma (FL), and Mantle B-Cell Lymphoma (MCL). DLBCL represents 30–40% of all adult NHL, making it the most common form [6]. Other categories of malignancies affecting B-cells include Chronic Lymphocytic Leukemia (CLL) and Small Lymphocytic Lymphoma (SLL). About 19,000 people are diagnosed with CLL/SLL in the United States each year. About 5–10% of patients with CLL undergo a “Richter’s transformation” in which they develop DLBCL during the course of illness [7]. BL, a high grade B cell malignancy, accounts for 3–4% of all lymphomas in adults in western countries although it occurs most frequently in children in areas with holo- and hyperendemic malaria and with less frequency in all other parts of the world [8]. The endemic form, typically presenting as tumors of the jaw in children, is strongly associated with Epstein-Barr Virus (EBV) and may also be associated with malaria. BL malignancies are rapidly growing, with a 24 h doubling time, and while treatments can be highly effective in children and adults (survival rates exceeding 70%), they are often much less effective for the elderly and immunocompromised [9–11]. These populations typically develop more severe treatment-associated toxicities from the aggressive treatment [12–14]. FL is the most common type of low grade NHL, is caused by the collection of abnormal B-cells in lymph nodes as follicles, and can often be managed with rituximab and chemotherapy. MCL is a rare but aggressive form of NHL that arises from the outer rim (mantle) of lymphoid cells. Although rituximab, an anti-CD20 monoclonal antibody, has been successfully used in conjunction with chemotherapy to treat several different types of lymphoma, the efficacy of its use in immunocompromised patients is controversial [15]. The aggressiveness of lymphoma subtypes and drawbacks to intensive chemotherapy treatment regimens highlight the importance of pursuing alternative immunotherapies for B-cell lymphomas founded on an improved understanding of the disease pathology.

In a normal immune response, HLA class I pathways activate CD8+ T cells to directly kill tumor cells. However, the CD8+ T cells do not survive long after killing the cells. In order to enhance tumor response efficacy, the CD8+ T cell survival and proliferation is supported by HLA class II activated CD4+ helper T cells [16,17]. In B-cell Lymphoma, HLA class 1 molecules, which typically activate CD8+ cytotoxic T cells (CTL) to directly kill tumors, are missing a key β-2 microglobulin subunit which results in immune escape for the tumor [18]. Additionally, research supports the finding that increased levels of IDO, Lag3, PD1, PDL, and TIM3 are all immunosuppressive to limit anti-tumor immunity [19]. Our laboratory found that BL and FL cells have deficiencies in the ability to effectively stimulate CD4+ T cells via the HLA class II pathway [8,20]. While co-stimulation was insufficient to generate a response, binding affinity for BL-associated class II molecules was confirmed, thus eliminating the possibility of faulty antigen/class II interaction as the source of the HLA class II pathway defect. However, subjecting BL cells to acidic conditions restored immune recognition via the formation of functional class II-peptide complexes. Analysis of the eluate indicated that the tumor-derived molecules may perturb antitumor immunity. Our laboratory has also shown that a membrane-bound enolase molecule is less abundant in BL tumors [20], which could be one of the possible mechanisms of immune escape of B-cell lymphoma. A number of recent studies have shown that an increased level of neuron specific enolase (NSE) is detected in DLBCL patients which may be linked with the poor prognosis of lymphoma patients and should be further explored in other lymphoid malignancies [21,22]. Thus, this editorial will also discuss the release of NSE by B-cell lymphoma and the possible mechanisms of immune escape by these tumors.

Enolase is a multifunctional enzyme abundantly expressed in the cytosol that typically functions in glucose metabolism by catalyzing the conversion of 2-phosphoglycerate to phosphoenolpyruvate [23–26]. Under inflammatory conditions, enolase can migrate from the cytosol to the cell surface where it enhances antigen presentation for host cell invasion via plasmin activation and subsequent extracellular matrix degradation leading to neurodegeneration [25]. This cell surface expression of enolase triggers the production of reactive oxygen species (ROS), nitric oxide (NO), and pro-inflammatory cytokines (TNF-α, IL-1β, IFN-γ, and TGF-β) and chemokines (MCP-1 and MIP-1α) to bolster neurodegenerative response [25,27]. Enolase levels are upregulated following neuronal injury in several conditions, and as such, the neuronal isoform of enolase, NSE, has been implicated as a biomarker of functional damages to neurons with prognostic value for acute spinal cord injury, traumatic brain injury, cardiac arrest, etc. [23,24,28]. There are three distinct, tissue-specific isoforms of enolase: α-enolase (non-neuronal enolase, ENO1), γ-enolase (NSE or ENO2), and β-enolase (muscle specific enolase, ENO3) [25,29]. During injury, ENO1, mostly found in adult tissues and present at the cell surface of B-cells, may trigger activating signals. ENO1 can be converted to NSE in neurons and cells of neuroendocrine origin and to ENO3 in muscle. NSE isoforms have also been found in microglia, oligodendrocytes, and astrocytes, indicating a connection between NSE expression and glial cell function [25,26]. However, it remains unclear why an increased level of NSE is detected in patients with B-cell lymphomas.

In general, B-cell lymphoma is not a neurodegenerative event. However, recent studies have shown increased serum NSE levels in various types of lymphoma correlating to disease progression or remission [6,22,30]. Elevated levels of serum NSE have been found in 17–21% of non-Hodgkin’s lymphoma and 6.5–23% of Hodgkin’s lymphoma patients [31]. Increased levels of NSE have also been detected in multiple myeloma, adult T-cell leukemia, melanoma, and neuroblastoma [26,30,32]. However, in these conditions, NSE may not be a specific marker, as a study investigating polyclonal NSE immunoreactivity in B and T cell malignant lymphoma (ML) found no correlation between reactivity and morphology or phenotype with inconsistencies even among B or T cell ML in 23 cases [33]. In acute lymphoblastic leukemia patients, serum NSE levels were closely associated with immunophenotype, risk stratification, and serum lactate dehydrogenase levels, indicating the prognostic value of NSE in some B-cell malignancies [30]. Recent studies have also indicated the connection between serum NSE and clinical outcome in DLBCL patients treated with rituximab-based chemo-immunotherapy [6,22]. After 4 cycles of therapy, 42 patients achieved remission, 6 achieved partial remission, and 5 remained stable or worse in terms of disease progression. Serum NSE levels significantly decreased over the course of treatment in remission patients while the levels increased or remained stable in those whose condition progressed accordingly. These studies also showed that NSE levels were an independent prognostic factor in the non-germinal center B-cell subtype of DLBCL, indicating that NSE may not only be a prognostic marker for disease progression but that it may also be involved in tumorigenesis.

**Figure 1:**
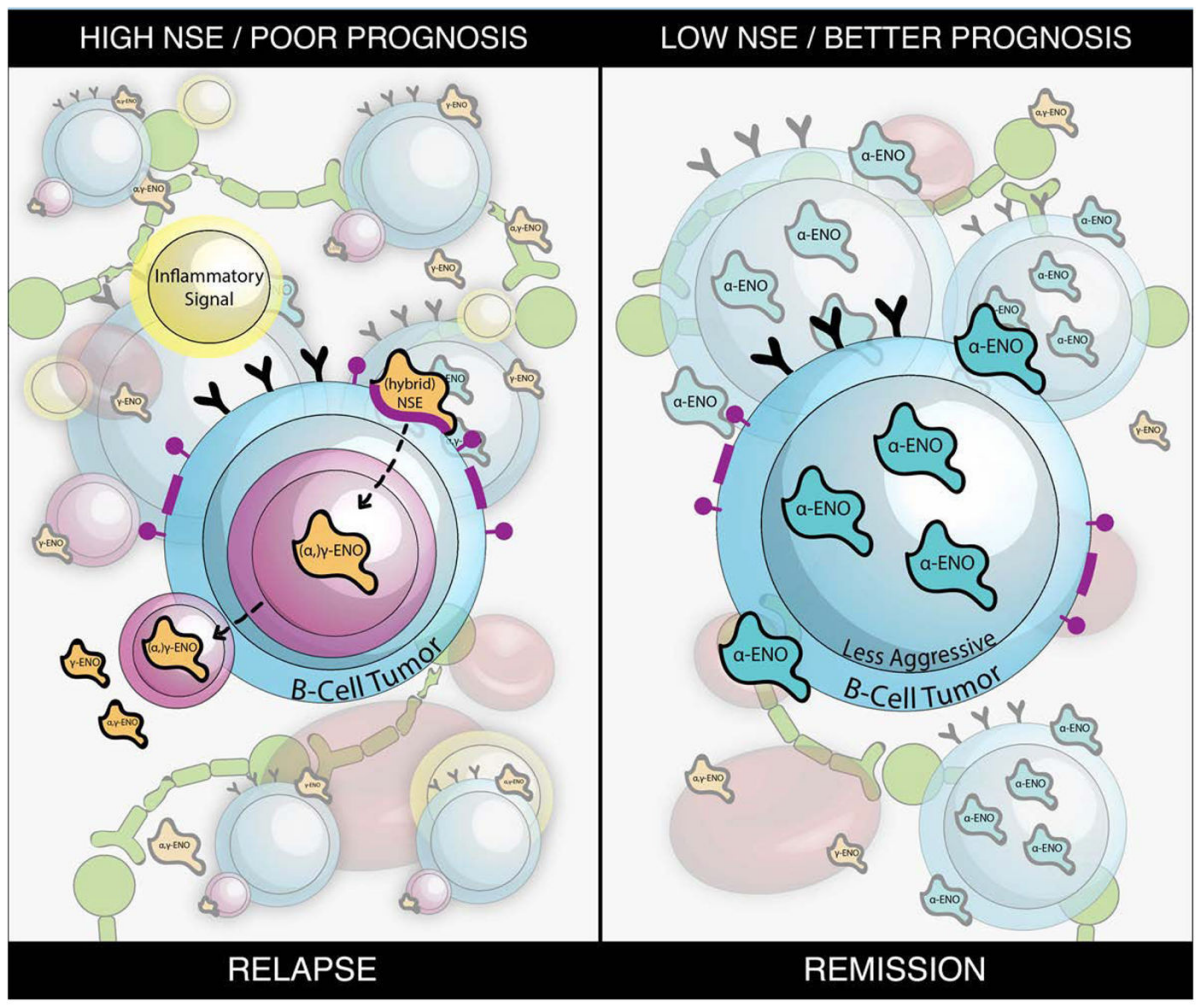
Potential role of NSE in immune escape and tumor progression in B-cell malignancies. Under inflammatory conditions, α-enolase can migrate to the cell surface of antigen presenting cells and lead to enhanced antigen presentation and production of inflammatory cytokines. This may promote inflammatory events such as neurodegeneration in elderly patients who are also suffering from malignant diseases like B-cell lymphoma. Increased detection of NSE in DLBCL patients are correlated with poor prognosis and typically seen in active cancer conditions. On the other hand, decreased levels of NSE are correlated with better prognosis or remission.

The association between lymphoma and NSE is interesting for several reasons. Firstly, NSE is generally thought to be specific to neurons and cells of neuroendocrine origin and used as a biomarker of neuronal injury. B-cell lymphoma pathology does not usually involve neuronal injury, yet NSE is detected in lymphoma patients. Therefore, there is a question of the increased serum NSE’s origin. One possibility is that late stage B-cell malignancies induce or enhance a base level neuronal injury and release of NSE that may be detectable in biological fluid. Perhaps an inflammatory pathway led to the development of B-cell lymphoma and is also linked to neuroinflammation or neuronal damage. Secondly, our study suggests that there is a membrane-bound form of α-enolase, which enhances antigen presentation and CD4+ T cell responses [20]. Though B-cells do not express NSE, they express α-enolase, which could be converted into NSE in neurons and ENO3 in muscle. It is possible that inflammation, which is characteristic of neurodegenerative conditions and lymphoma, induces this conversion of ENO1 into a hybrid form of NSE (αγ NSE) via a pro-inflammatory pathway involving activated B-cells (Figure 1). It remains unclear if Bcell lymphomas secrete α-enolase and apply mechanisms to escape T cell recognition. However, cell surface expression of enolase is reduced in BL malignancies [20] which both supports our hypothesis and suggests a possible mechanism for immune escape by B-cell tumors. We further hypothesize that the membrane-bound form of enolase could be providing activating signals while the secreted form does not, as the secreted form is linked with poor prognosis. It is possible that in cancer or inflammatory conditions there could be a secretory pathway that allows for the conversion of ENO1 into a hybrid form of NSE such as αγ-NSE or just γ-NSE in neurons, and subsequent secretion into serum or plasma. B-cell tumors may capture NSE through receptor mediated endocytosis and release it in advanced stages of the disease (Figure 1). This secreted form of NSE may also apply multiple unknown mechanisms to attenuate antitumor immunity in lymphoma patients.

Based on the current understanding of enolase expression in lymphoma and inflammatory conditions, we believe that exploration of enolase conversion could illuminate the missing link between inflammation, cancer and neuronal injury in lymphoid malignancies. This possibility is one that warrants further investigation to augment our understanding of the increasingly complex pathophysiological mechanisms of B-cell lymphoma. With a clearer conception of the connection between inflammation and NSE in lymphoma, we may find new biomarkers as therapeutic targets for better treatment strategies.

## References

[1] Siegel RL, Miller KD, Jemal A (2018). Cancer statistics, 2018. CA Cancer J Clin.

[2] Cannon AC, Loberiza FR (2015). Review of antibody-based immunotherapy in the treatment of non-Hodgkin lymphoma and patterns of use. Clin Lymphoma Myeloma Leuk.

[3] Amria S, Cameron C, Stuart R, Haque A (2008). Defects in HLA class II antigen presentation in B-cell lymphomas. Leuk Lymphoma.

[4] Armitage JO, Gascoyne RD, Lunning MA, Cavalli F (2017). Non-Hodgkin lymphoma. Lancet.

[5] Kuppers R, Klein U, Hansmann ML, Rajewsky K (1999). Cellular origin of human B-cell lymphomas. N Engl J Med.

[6] Wang L, Liu P, Chen X, Geng Q, Lu Y (2012). Serum neuron-specific enolase is correlated with clinical outcome of patients with non-germinal center B cell-like subtype of diffuse large B-cell lymphoma treated with rituximab-based immunochemotherapy. Med Oncol.

[7] Jain P, O’Brien S (2012). Richter’s transformation in chronic lymphocytic leukemia. Oncology (Williston Park).

[8] God JM, Haque A (2016). Multiple Defects Impair the HLA Class II Antigen Presentation Capacity of Burkitt Lymphoma. J Clin Cell Immunol.

[9] God JM, Haque A (2011). Immune Evasion by B-cell Lymphoma. J Clin Cell Immunol.

[10] Di Nicola M, Carlo-Stella C, Mariotti J, Devizzi L, Massimino M (2004). High response rate and manageable toxicity with an intensive, short-term chemotherapy programme for Burkitt’s lymphoma in adults. Br J Haematol.

[11] Divine M, Casassus P, Koscielny S, Bosq J, Sebban C (2005). Burkitt lymphoma in adults: a prospective study of 72 patients treated with an adapted pediatric LMB protocol. Ann Oncol.

[12] De Leval L, Hasserjian Rp (2009). Diffuse Large B-Cell Lymphomas And Burkitt Lymphoma. Hematol Oncol Clin North Am.

[13] Noy A (2010). Controversies in the treatment of Burkitt lymphoma in AIDS. Curr Opin Oncol.

[14] Oriol A, Ribera JM, Bergua J, Gimenez Mesa E, Grande C (2008). High-dose chemotherapy and immunotherapy in adult Burkitt lymphoma: comparison of results in human immunodeficiency virus-infected and noninfected patients. Cancer.

[15] Blinder VS, Chadburn A, Furman RR, Mathew S, Leonard JP (2008). Improving outcomes for patients with Burkitt lymphoma and HIV. AIDS Patient Care STDS.

[16] Bou Nasser Eddine F, Ramia E, Tosi G, Forlani G, Accolla RS (2017). Tumor Immunology meets…Immunology: Modified cancer cells as professional APC for priming naive tumor-specific CD4+ T cells. Oncoimmunology.

[17] Godfrey DI, Le Nours J, Andrews DM, Uldrich AP, Rossjohn J (2017). Unconventional T Cell Targets for Cancer Immunotherapy. Immunity.

[18] Jordanova ES, Riemersma SA, Philippo K, Schuuring E, Kluin PM (2003). Beta2-microglobulin aberrations in diffuse large B-cell lymphoma of the testis and the central nervous system. Int J cancer.

[19] Capone M, Bryant JM, Sutkowski N, Haque A (2016). Fc Receptor-Like Proteins in Pathophysiology of B-cell Disorder. J Clin Cell Immunol.

[20] God JM, Zhao D, Cameron CA, Amria S, Bethard JR (2014). Disruption of HLA class II antigen presentation in Burkitt lymphoma: implication of a 47,000 MW acid labile protein in CD4+ T-cell recognition. Immunol.

[21] Mohammad RM, Hamdan MY, al-Katib A (1994). Induced expression of alpha-enolase in differentiated diffuse large cell lymphoma. Enzyme Protein.

[22] Wang L, Liu P, Geng Q, Chen X, Lv Y (2011). Prognostic significance of neuron-specific enolase in patients with diffuse large B-cell lymphoma treated with rituximab-based immunochemotherapy. Leuk Lymphoma.

[23] Hafner A, Obermajer N, Kos J (2012). Gamma-Enolase C-terminal peptide promotes cell survival and neurite outgrowth by activation of the PI3K/Akt and MAPK/ERK signalling pathways. Biochem J.

[24] Haque A, Capone M, Matzelle D, Cox A, Banik NL (2017). Targeting Enolase in Reducing Secondary Damage in Acute Spinal Cord Injury in Rats. Neurochem Res.

[25] Haque A, Ray SK, Cox A, Banik NL (2016). Neuron specific enolase: a promising therapeutic target in acute spinal cord injury. Metab Brain Dis.

[26] Vizin T, Kos J (2015). Gamma-enolase: a well-known tumour marker, with a less-known role in cancer. Radiol Oncol.

[27] Hafner A, Glavan G, Obermajer N, Zivin M, Schliebs R (2013). Neuroprotective role of gamma-enolase in microglia in a mouse model of Alzheimer’s disease is regulated by cathepsin X. Aging Cell.

[28] Li M, Wen H, Yan Z, Ding T, Long L (2014). Temporal-spatial expression of ENOLASE after acute spinal cord injury in adult rats. Neurosci Res.

[29] Haque A, Polcyn R, Matzelle D, Banik NL (2018). New Insights into the Role of Neuron-Specific Enolase in Neuro-Inflammation, Neurodegeneration, and Neuroprotection. Brain Sci.

[30] Liu CC, Wang H, Wang JH, Wang L, Geng QR (2016). Serum neuron-specific enolase levels are upregulated in patients with acute lymphoblastic leukemia and are predictive of prognosis. Oncotarget.

[31] Nakatsuka S, Nishiu M, Tomita Y, Miwa H, Takakuwa T (2002). Enhanced expression of neuron-specific enolase (NSE) in pyothoraxassociated lymphoma (PAL). Jpn J Cancer Res.

[32] Yang H, Mi R, Wang Q, Wei X, Yin Q (2014). Expression of neuron-specific enolase in multiple myeloma and implications for clinical diagnosis and treatment. PLoS One.

[33] Nemeth J, Galian A, Mikol J, Cochand-Priollet B, Wassef M (1987). Neuron-specific enolase and malignant lymphomas (23 cases). Virchows Arch A Pathol Anat Histopathol.

